# Comparison of Services of Public, Private and Foreign Hospitals from the Perspective of Bangladeshi Patients

**Published:** 2007-06

**Authors:** Nazlee Siddiqui, Shahjahan Ali Khandaker

**Affiliations:** 1 School of Business, North South University, 12 Kemal Ataturk Avenue, Banani, Dhaka 1213, Bangladesh; 2 Health Economics Unit, Ministry of Health and Family Welfare, Government of Bangladesh, Dhaka 1000, Bangladesh

**Keywords:** Comparative studies, Foreign Hospitals, Healthcare, Health services, Private Hospitals, Public Hospitals, Quality of services, Bangladesh

## Abstract

Despite recent developments in the Bangladesh healthcare sector, there is still great concern about the quality of healthcare services in the country. This study compared the quality of healthcare services by different types of institutions, i.e. public and private hospitals, from the perspective of Bangladeshi patients to identify the relevant areas for development. A survey was conducted among Bangladeshi citizens who were in-patients in public or private hospitals in Dhaka city or in hospitals abroad within the last one year. About 400 exit-interviews were conducted using a structured questionnaire that addressed the probable factors of the quality of healthcare services in 5-point interval scales. The results gave an overview of the perspectives of Bangladeshi patients on the quality of service in three types of hospitals. The quality of service in private hospitals scored higher than that in public hospitals for nursing care, tangible hospital matters, i.e. cleanliness, supply of utilities, and availability of drugs. The overall quality of service was better in the foreign hospitals compared to that in the private hospitals in Bangladesh in all factors, even the ‘perceived cost’ factor. This paper provides insights into the specific factors of the quality of hospital services that need to be addressed to meet the needs of Bangladeshi patients.

## INTRODUCTION

Bangladesh has a good healthcare network covering both rural and urban areas. There are 3,976 healthcare facilities in the public sector and 975 privately-run hospitals/clinics (1). The healthcare-delivery system of the country compares favourably with that of many other Asian countries. However, overall healthcare use/consumption in Bangladesh is low and is of great concern to society. A survey by the Centre for International Epidemiological Training (CIET), Canada, showed that, in Bangladesh, 13% of treatment-seekers use government services, 27% use private/NGO services, and 60% unqualified services (2). In their comparative survey on private and public healthcare providers in Bangladesh, Ricardo et al. observed that the overall use-rate for public healthcare services was as low as 30% (3). On the other hand, the uneven distribution of demand is creating unmanageable pressure on the few reputable public hospitals. In their ‘Bangladesh healthcare facility efficiency study’, Ranan and Somanthan observed that the overall patient load in public medical college hospitals was approximately five times higher than that in other general hospitals (4). A study conducted by the Health Economics Unit (HEU) of the Ministry of Health and Family Welfare (MoHFW), Government of Bangladesh, found that the unavailability of doctors and nurses, their attitudes and behaviour, lack of drugs, waiting time, travel time, etc. contributed to the low use of public hospitals (5).

In response to the growing disappointment in the role of the public healthcare sector, the number of private-run facilities has increased. An estimated 15% growth has been observed between 1996 and 2000 in this sector (1). However, quali-ty is a major concern both in public and private healthcare services. Such concern is prompting a large number of Bangladeshi patients to seek foreign medical care despite the additional costs, travel, lengthy visa procedures, etc. According to the official record of the Institute of Health Economics, University of Dhaka, Bangladeshis spend approximately Tk 500 million a year on foreign healthcare services (6).

The quality of service in general is of inherent importance in any society. In the late 1980s, Parasuraman et al. presented some parameters of the quality of service, i.e. reliability, responsiveness, assurance, tangibility, and empathy (7). Andaleeb introduced some more Bangladesh market-specific service-quality parameters, i.e. baksheesh and discipline (8,9). Various reports from the HEU presented further service-quality parameters, such as access cost of healthcare services (5,10). Aldana et al. and Rahman et al. analyzed the quality of Bangladesh healthcare services from the perspective of patients (11,12). However, these studies were limited to some extent, as they did not cover the experiences of Bangladeshi patients with foreign healthcare services with an evidence-based case. The present study compared the quality of healthcare services between in-country public and private hospitals and hospitals abroad from the pers-pective of Bangladeshi patients. Based on these findings, some recommendations have been made to improve in-country healthcare services to meet the needs of Bangladeshi patients

## MATERIALS AND METHODS

### Background

Quality of service is an elusive and indistinct construct and is difficult to measure. Perceptions of the quality of service result from a comparison of the expectations of consumers with the performance of actual services. Evaluation of the quality of service is not made solely on the outcome of a service rather it also involves evaluation of the process of service-delivery (13). Certain criteria that measure the quality of service should be identified first, and then an analysis of those identified criteria would definitely provide a systematic assessment of the quality of service (8). Parasuraman et al. developed the SERVQUAL model along these lines to measure the quality of service (7,13,14). However, this model and its measurement are not beyond criticism, as it cannot measure quality in all service sectors (8).

The present study used the SERVQUAL framework refined by Andaleeb (8), as this is more suitable for the Bangladesh perspective. Some additions were made to the framework of Andaleeb (8) resulting from the two sessions of focus-group discussions (FGDs) conducted for this study. Each focus group comprised eight members, who had used healthcare services in Dhaka or abroad within the last one year. These members were selected on a random basis without any restriction on gender, occupation, etc. It was ensured that each group had a mixture of in-country and foreign hospital patients. The ‘perceived cost’ variable focused on the cost of laboratory tests is an example of an addition to the research tool as a result of FGD feedback.

The initial nine variables—accessibility, reliability, tangibility, responsiveness, assurance, communication, empathy, perceived cost, and discipline of the research tool—were run through factor analysis to arrive at the final eight variables—empathy of physicians, availability of physicians, assurance/competence of physicians, empathy of nurses, responsiveness of nurses, availability of drugs, tangibility, and perceived cost. Descriptions of these variables are provided in the Appendix. Testing of the hypothesis was also done using these variables. Statements of the hypotheses are given below:

- The empathy of physicians, availability of physicians, assurance/competence of physicians, empathy of nurses, responsiveness of nurses, availability of drugs, tangibility, and perceived cost at in-country public and private healthcare services are the same.
- The empathy of physicians, availability of physicians, assurance/ competence of physicians, empathy of nurses, responsiveness of nurses, availability of drugs, tangibility, and perceived cost at private healthcare services in Bangladesh and healthcare services abroad are the same.

### Questionnaire design

A preliminary questionnaire was developed in English in line with the constructs of the hypotheses. This was translated into Bangla at the final stage. The 5-point interval scales were used in the structured format with verbal statements, such as ‘strongly disagree’ and ‘strongly agree’, anchored to the numerals of 1 to 5. The SERVQUAL scales of Parasuraman et al. were 7-point interval scales (7). In this study, a 5-point interval scale made better sense considering the education and exposure of the sample base. Multiple items were used for representing each construct. Researchers pretested the questionnaire and adopted it accordingly. In pretesting, 10 interviews were conducted: seven with in-patients in Bangladesh hospitals (private and public) and three with Bangladeshi patients who received healthcare services from hospitals in Bangkok, Thailand, within the last one year.

As per internationally-accepted ethical practice, the questionnaire mentioned that the survey would not require respondents to provide their personal details and that data provided by respondents would be dealt with confidentially. It was also mentioned that, while respondents are encouraged to answer all the questions in the questionnaire, they could refrain from answering any specific question or even quit the survey as and when they desired.

### Data-collection method

A 10-member data-collection team was recruited from final year Bachelors in Business Administration (BBA) students of East-West University, Dhaka. Researchers trained data collectors on interview techniques to minimize bias. Permission from the MoHFW was obtained to facilitate the data-collection process.

### Sampling method

Two separate lists of public and private hospitals in Dhaka along with the bed capacity of relevant hospitals were obtained from the MoHFW. Two public hospitals—Pub 1 and 2—were chosen purposively considering a similar patient load. Three private hospitals—Pri 1, Pri 2, and Pri 3—were randomly selected from the list of private hospitals.

In the case of surveys at public and private hospitals in Bangladesh, the data collectors interviewed in-patients on the hospital premises. At the hospitals, respondents were initially identified from the list of ‘to be released patients’ provided by the hospital authority. The data collectors randomly selected samples from this list and obtained data as per the prescribed questionnaire. In cases where the approached patients were not interested in participating in the survey, the data collectors moved to another patient on the list.

Data for patients using foreign hospital services were difficult to collect in a probability sampling method, as there were no sources to provide complete information on such flow of patients. As a result, the snowball sampling method, a non-probability technique of drawing samples based on referral from the initial respondent, was used for collecting data through students of East-West University, North South University, and the University of Liberal Arts, Dhaka. Respondents were screened for the same eligibility criteria of being an in-patient at a foreign hospital within the last one year.

### Sample size

Due to resource and time constraints, a maximum of 450 samples was planned for the study. About 150 samples were to be collected from each stratum of public, private, and foreign hospitals. The in-country hospital sample base was Dhaka city as Dhaka hosts different qualities of hospitals and the highest number of hospitals in the country.

Table 1 shows the break-up of the actual 400 samples collected in this study.

**Table 1:** Break-up of 400 study samples

Private patients (n=153)			Public patients (=153)		Foreign patients (n=94)			
Pri 1	Pri 2	Pri 3	Pub 1	Pub 2	India	Thailand	Single	Others
65	52	36	105	48	69	16	7	1

The sample size of 400 is consistent with the intended sample size value that could be calculated assuming 50% population proportion (p) with a 95% confidence interval (corresponding z value of 1.96) and sampling error level of 5% (e value). Therefore, this study assumed that 50% of the population in Bangladesh could express knowledge on the quality of hospital services, and the true population value would be within plus or minus 5% of the estimates based on this sample. As per the formula, n=z^2^[p(1-p)]/e^2^; [(1.96)^2^ ∗ (0.5 ∗ 0.5)]/(0.05)^2^=384, which is near to 400 (15).

### Analysis

As per the results of the factor analysis and reliability check, the service-quality variables were finalized to be: availability of physicians, assurance/competence of physicians, empathy of physicians, responsiveness of nurses, empathy of nurses, availability of drugs, perceived cost of healthcare service, and overall tangibility of the healthcare service centre.

In factor analysis, scales having a loading value of 0.5 and higher were accepted as an important component of the variable. Each factor was analyzed using Kaiser's eigen value of greater than or equal to one (16) to see whether each component measured a single factor or not. Lastly, the factors forming a component were tested for reliability (alpha=0.6 or higher). Following this technique, the above-mentioned eight variables of the quality of healthcare services were obtained.

A mean comparison on the identified healthcare service-quality variables was done through independent sample t-test in two phases. At one phase, comparisons were done between the in-country private hospitals and the public hospitals. The other phase covered the comparisons between the private hospitals in Bangladesh and the foreign hospitals.

## RESULTS

The findings of this study showed a significant quality gap in healthcare services both within the in-country group and the group of private and foreign healthcare providers. Table 2 shows the quality of private healthcare services scoring better in the factors of availability of drugs, tangibility, perceived costs, empathy of nurses, and responsiveness. Table 3 shows that the quality of service of foreign hospitals is better in all the factors compared to that of private hospitals in Bangladesh.

**Table 2:** Independent samples *t*-test for public vs private hospitals

Variable		*t*-test for equality of means		
	*t*	df	Significance (2-tailed)	Mean difference
Availability of physicians	−1.616	304	0.107	−0.1291
	−1.616	279.771	0.107	−0.1291
Assurance and competence of physicians	−0.534	304	0.593	−0.0433
	−0.534	303.338	0.593	−0.0433
Empathy of physicians	−1.692	304	0.092	−0.1425
	−1.692	288.179	0.092	−0.1425
Empathy of nurses	−3.164	304	0.002	−0.2936
	−3.164	297.538	0.002	−0.2936
Responsiveness of nurses	−2.143	304	0.033	−0.1850
	−2.143	280.299	0.033	−0.1850
Tangible	−10.652	304	0.000	−0.7694
	−10.652	294.143	0.000	−0.7694
Drug	−11.447	303	0.000	−0.9915
	−11.438	285.077	0.000	−0.9915
Perceived cost	−7.304	302	0.000	−0.6151
	−7.304	301.762	0.000	−0.6151

df=Degrees of freedom

**Table 3:** Independent samples *t*-test for private hospitals vs foreign hospitals

Variable	*t*-test for equality of means			
	*t*	df	Significance (2-tailed)	Mean difference
Availability of physicians	−7.960	245	0.000	−0.5734
	−8.338	225.104		−0.5734
Assurance and competence of physicians	−7.621	245	0.000	−0.6228
	−8.255	240.102		−0.6228
Empathy of physicians	−7.452	245	0.000	−0.5947
	−7.753	221.295		−0.5947
Empathy of nurses	−4.388	245	0.000	−0.3995
	−4.634	229.380		−0.3995
Responsiveness of nurses	−7.680	245	0.000	−0.5876
	−8.175	233.200		−0.5876
Tangible	−9.764	245	0.000	−0.6665
	−10.454	235.760		−0.6665
Drug	−7.074	245	0.000	−0.5736
	−7.383	223.114		−0.5736
Perceived cost	4.972	245	0.000	0.5343
	4.723	165.658	0.000	0.5343

df=Degrees of freedom

The factor of ‘availability of drugs’ showed the most significant difference between these two healthcare service providers. This is demonstrated by the t value of −11.447 and the mean difference of −0.9915. Therefore, the service of public hospitals in relation to availability of drugs and timely administration to patients was poorer than that of the private hospitals.

The public hospitals seem to be in a much worse condition compared to the private hospitals regarding cleanliness of the hospital, water supply, availability of equipment, etc. The ‘tangibility’ factor, with the t value of −10.652 and the mean difference of −0.769, conveys this point.

The results also showed that the ‘perceived cost’ at the private hospital was significantly higher (i.e. t value of −7.3 and the mean difference of −0.615) than that of the public hospitals. The ‘cost’ factor was measured not as the absolute amount paid but as the patients’ perceptions of the reasonability of the cost paid for the consultation of a physician, diagnostics, accommodation, etc.

With regard to nurse-related matters—empathy and responsiveness of nurses—the quality of the public healthcare service provider also seemed to be much worse than that of the private hospitals. These are demonstrated through the corresponding t value of −3.164 and −2.143 accordingly.

It is interesting to note that regarding physician-related matters—availability, empathy, and assurance/competence of physicians—there were no significant differences between the quality of services in the public and private hospitals.

General cleanliness of toilets, cabins, water supply, etc. were much better in the foreign hospitals compared to the private hospitals in our country. The t value of −10.45 in the factor of ‘tangibility’ demonstrated this point. Tangibility—the amenities of care—is the factor that scored the highest service gap between these two types of hospitals.

The findings also showed that Bangladeshi patients found physicians in the foreign hospitals more available, competent, and empathetic compared to those in the private hospitals in Bangladesh. Table 3 shows that, regarding physician-relevant matters, i.e. assurance/competence, availability and empathy of physicians, there were substantial mean differences, being close to −0.6 or higher in favour of the foreign hospitals.

While the nurses in the foreign hospitals attained a better service rating in general, the service-quality gap in the responsiveness (i.e. mean difference of −0.5876) was higher than that of the empathy factor (the mean difference of −0.3995) compared to the nurses of the Bangladeshi private hospitals.

Another important factor in the service-quality gap between the Bangladeshi private hospitals and the foreign ones was availability of drugs with a mean difference of −0.5736 and t value of −7.383. The Bangladeshi patients found the services of foreign hospitals in availability of drugs and timely administration to patients to be much better than that of the private hospitals in Bangladesh.

The mean of costs of factors, such as consultation of physician, operation, diagnostics, etc., were found to be unreasonable in the private hospitals of Bangladesh compared to the public and foreign hospitals. In this study, 50% of the in-country private hospital patients and foreign hospital patients (mostly Indian hospital patients) belonged to the same income range. Also, the patients in the foreign hospitals paid higher costs than the private hospitals in absolute terms. Despite this, the Bangladeshi patients perceived foreign healthcare services to be more reasonably priced.

## DISCUSSION

This study analyzed the service quality of in-country public and private hospitals from the perspective of Bangladeshi patients in an exhaustive manner. Comparison of public and private hospitals in Bangladesh with foreign hospitals derived from this study should allow healthcare service providers to make provision for improving the quality of services of the in-country hospitals.

Tangibility (Item 8 in Appendix) came out to be a general weakness in the Bangladesh healthcare sector. In this study, ‘tangibility’ did not cover cleanliness of service providers (i.e. doctors, nurses), but all other common issues, such as supply of utilities, cleanliness of hospital (toilets/cabins), and condition of equipment, were covered. The World Health Organization (WHO) had identified that about 50% of the medical equipment in developing countries is unusable (17). The importance of tangibility matters in the Bangladesh healthcare sector has also been earlier discussed. It has been shown that improvement in tangibility matters enables better service-delivery and results in improved use of healthcare facilities (18). The recent establishments in the Bangladesh private healthcare sector; i.e. Apollo and Shikder, are some initiatives in this regard. From the initial stage, service providers should emphasize maintenance of such standards of tangibility on a long-term basis. The scope of the private sector's cooperation in improving the tangibility matters of the existing public hospitals could be worth exploring.

This study confirmed the general opinion that the quality of service of physicians in terms of availability, assurance/competency, and empathy (Items 2 and 3 in Appendix) in the Bangladeshi hospitals are poor compared to foreign hospitals. This is consistent with the findings of Ricardo et al. that 100% of outpatients at public hospitals and 47% of the same at private hospitals are not attended to at the appointed time (3). Reputable physicians in our country are known to shuffle themselves between different hospitals visiting an unreasonable number of patients each day, which makes them totally incapable of allowing due time and assurance/competency to patients (19). In his ethnographic study at a public hospital, Zaman gave evidence of physicians leaving public hospitals early for private practice (20). The healthcare service providers in our country should initiate continuous technical and behavioural training and an evaluation programme for physicians. According to the World Bank, there is little documented evaluation on the quality of care by physicians in Bangladesh, with regard to both public sector and private sector (21). The practice of limiting the maximum patients to be visited by a physician a day could be implemented both at public hospitals and private hospitals. Research should be done to reveal whether such measures could add enough value in patients’ mind to outweigh relevant cost increases.

This study has shown that the service-quality gap between nurses of private hospitals and those of foreign hospitals in terms of empathy and responsibility (Items 4 and 5 in Appendix) is much higher than the similar service-quality gap that exists between nurses of private and public hospitals. Therefore, the study could bring out the reality that, while the nurses in private hospitals are doing a better job than their counterparts in public hospitals, they still lacking compared with the service level of nurses in the foreign hospitals. Concerns with services of nurses in Bangladesh were also raised in previous studies. Patients in Bangladesh are very dissatisfied with the behaviour of nurses and inefficiency in service-delivery (12). Results of a study revealed that nurses in Bangladesh decline to provide direct patient care due to sociocultural barriers (22). In the same study, it was identified that nurses in general also suffer from inferior social status. This study does not provide any individual focus on probable improvement measures for nurses. However, observing healthcare services in the private sector in Bangladesh and abroad, it could be understood that better package, availability of equipment, and strict supervision of management that allows development of nurses as well would definitely contribute to improve services of nurses. Bangladesh and its development partners, i.e. World Bank, WHO, and Department for International Development, have been taking steps to develop skills and leadership of nurses (22).

The findings of this study explain that the quali-ty gap in services regarding drug issue to be much higher in the case of the public vs private scenario compared to the private vs foreign scenario. The availability of drugs and their timely administration (Item 6 in Appendix) to patients are an important factor of the quality of service as patients draw a logical relationship between drugs and the curing process of a disease. Policy-makers need to address mismanagement of drugs especially in public hospitals in Bangladesh. Patients at public hospitals face great difficulties in getting prescribed medicines and ultimately resort to buying medicines from outside. Inadequate supplies of drugs/medicines deter nursing services (22), while the inefficient management of drugs could waste scarce resources as well (23). Although the situation in private hospitals is much better in this regard, they still lack the kind of comprehensive medicine management process as followed in foreign hospitals. While the process of management of the drugs in the foreign hospitals could be the benchmark to follow for well-resourced private hospitals, outsourcing of the public hospitals to the private sector may ensure a non-profit but efficient management. Some Indian public hospitals have been using non-governmental organizations (NGOs) for running a non-profit pharmacy (23); these experiences could be used in our case.

Perceived cost of private healthcare services (Appendix) came out to be more unreasonable than that of foreign healthcare services. This result could indicate the existence of price and quality imbalance in the Bangladeshi healthcare sector. As stated in the Results section in this study, Bangladeshis within a similar income-range used in-country private health facilities and foreign hospitals Therefore, all such findings might be an indication for private hospitals in Bangladesh to maintain a quality focus even at the option of increasing the cost. However, further studies should be done to clarify the issue. It should be noted that the present study could reveal such unusual findings regarding ‘perceived cost’, as respondents were asked about the cost of the whole process: cost of service providers, accommodation, travelling, etc. Hence, there is an apparent need for private hospitals to maintain higher service quality throughout the process chain: service of physicians, service of nurses, management of drugs, tangibility, etc.

Since this study indicates, the perceived cost of private hospitals is significantly higher than that of public hospitals in the minds of Bangladeshi patients. It is our social obligation to remember that the high-cost of private healthcare services cannot be suitable for a greater part of our needy population. The Government of Bangladesh has taken account of possible private-public cooperation, and various studies confirmed the potential of contracting out some public healthcare services to the private sector (24,25). Policies to have a higher number of mandatory free treatments in affluent hospitals and certain profit contribution from profit-making private hospitals for the development of public hospitals might be researched in the interest of the majority of the population.

## Limitations

This study has several limitations, i.e. Dhakabased study as opposed to a national one, and snowball sampling method for foreign patients as opposed to a probability sampling method. A bigger sample size covering more hospitals in the country could have made the paper more robust. In future, opinions of service providers and policy-makers should be sought on the factors where a service-quality gap was found and also on the recommendations that have been discussed in this study. Such work would help draw proper benefit from this study.

The study attempted to identify the specific factors of the quality of service in the Bangladesh healthcare sector that should be improved from the perspectives of Bangladeshi patients. This comparative study has shown that availability of physicians, assurance/competence of physicians, empathy of physicians, responsiveness of nurses, empathy of nurses, availability of drugs, tangibility (amenities of care), and perceived cost were certain factors in which significant differences existed among the public, private and foreign hospitals. The respective service providers should, thus, undertake necessary steps to mitigate this gap to offer better healthcare services to patients. Such steps would contribute to the sustainable growth of the healthcare sector in Bangladesh.

The suggested key recommendations in this study are: continuous training and evaluation of physicians and nurses, a holistic quality focus throughout the service process in private hospitals, cooperation between private and public hospitals in areas of availability of drugs, tangibility/amenities of care, and various healthcare-development projects.

## Appendix: Variables determining the quality of healthcare services

1. Physicians: empathy
  - Doctor was sincere whenever necessary
  - Doctor was willing to answer any question
  - Doctor listened to you attentively
  - Doctor rightly referred to your previous problems
  - Doctor was consistently caring
2. Physicians: availability
  - Doctors and specialists were available when required
  - Doctors followed up treatments regularly
  - Doctors were present during visiting hours
3. Physicians: assurance/competence
  - Doctors interpreted laboratory reports correctly
  - Doctor gave correct treatment at the first time
  - Doctors were competent in diagnosing the problem
  - Doctors gave knowledgeable answers to questions
  - You felt safe in the hands of the doctors
4. Nurses: empathy
  - Nurse communicated your problem to doctors
  - Nurse understood your problem
  - Nurse explained prescription to patient/ relatives
  - Nurse was consistently caring
  - Nurse paid individual attention to patient
  - Nurse provided moral courage
5. Nurses: responsiveness
  - Nurse administered treatment in time
  - Nurse was willing to respond to patients' call
  - Nurse cared patient cordially whenever called
  - Nurse replied correctly to patients query
  - You felt comfort with nurse service
6. Drugs
  - Drug was available 24 hours at premises
  - Prescribed drug was timely supplied to patient
  - Nurses administered drugs to patients with own hand
7. Perceived cost
  - Doctor's consultation fee was higher
  - Laboratory test fee was higher
  - Operation cost was higher
  - Travel cost was higher
  - Accommodation cost was higher
8. Tangibility
  - Hospital was visually appealing
  - Hospital premises were neat and clean
  - There was enough waiting room/space
  - Healthcare centres had modern equipment
  - Cabin/Ward's bedding and floors were clean
  - Cabin/Ward's waste bins were regularly cleaned
  - Hospital had regular water supply
  - Hospital had regular electricity
  - Hospital had adequate security
  - Toilets and bathrooms were clean
